# Comorbid anorexia nervosa and schizophrenia

**DOI:** 10.1192/j.eurpsy.2021.653

**Published:** 2021-08-13

**Authors:** C. De Andrés Lobo, C. Vallecillo Adame, T. Jiménez Aparicio, M. Queipo De Llano De La Viuda, A. Gonzaga Ramírez, G. Guerra Valera, I. Santos Carrasco, J. Gonçalves Cerejeira, C. Capella Meseguer, E. Rodríguez Vázquez

**Affiliations:** 1 Psiquiatría, Hospital Clínico Universitario de Valladolid, Valladolid, Spain; 2 Psiquiatría, Hospital Clínico Universitario Valldolid, Valladolid, Spain; 3 Psiquiatría, Hospital Clínico Universitario Valladolid, Valladolid, Spain; 4 Psiquiatría, HCUV, Valladolid, Spain

**Keywords:** anorexia nervosa, body image, schizophrénia, eating disorder

## Abstract

**Introduction:**

Although schizophrenia and anorexia nervosa are very different disorders, when they occur in the same patient it can be difficult to distinguish whether the alterations in body image are due to psychotic symptoms or correspond to a comorbid eating disorder. It is also relevant to know how they can interact with each other.

**Objectives:**

Presentation of a clinical case of anorexia nervosa in the context of a patient with a previous diagnosis of paranoid schizophrenia.

**Methods:**

Bibliographic review of the relationship between schizophrenia and alterations in the perception of body image by searching for articles in Pubmed.

**Results:**

We present a 48-year-old woman who resides with her mother and a sister. Diagnosed with paranoid schizophrenia and eating disorder. She had previously been admitted to hospital twice. Since 2004, she has been followed up in mental health consultations. The patient reports constant weight changes. A year ago she began to feel overweight and began to restrict her intake and to exercise, having lost 20 kg. She reports psychotic symptoms in the past, that she now denies. Various scales show moderate impact of weight on personal perception of psychosocial adjustment, an impulse to thinness and a significant distortion of body image, perceiving herself as heavier than she is and wishing she was lighter.

**Conclusions:**

In schizophrenia, confusion in bodily experiences and states is not uncommon. The possible interactions between the symptoms of schizophrenia and anorexia nervosa complicate the adequate care of these patients. Further research on comorbidity of these two disorders is necessary.

